# Incretin triple agonist retatrutide (LY3437943) alleviates obesity-associated cancer progression

**DOI:** 10.1038/s44324-025-00054-5

**Published:** 2025-03-14

**Authors:** Sandesh J. Marathe, Emily W. Grey, Margaret S. Bohm, Sydney C. Joseph, Arvind V. Ramesh, Matthew A. Cottam, Kamran Idrees, Kathryn E. Wellen, Alyssa H. Hasty, Jeffrey C. Rathmell, Liza Makowski

**Affiliations:** 1 Department of Medicine, Division of Hematology and Oncology, College of Medicine, The University of Tennessee Health Science Center, Memphis, TN USA; 2 UTHSC Center for Cancer Research, College of Medicine, The University of Tennessee Health Science Center, Memphis, TN USA; 3 Department of Microbiology, Immunology, and Biochemistry, College of Medicine, The University of Tennessee Health Science Center, Memphis, TN USA; 4 Division of Surgical Oncology, Department of Surgery, Vanderbilt University Medical Center, Nashville, TN USA; 5 Department of Cancer Biology, Perelman School of Medicine, University of Pennsylvania, Philadelphia, PA USA; 6 Department of Internal Medicine, Touchstone Diabetes Center, UT Southwestern, Dallas, TX USA; 7 Vanderbilt Center for Immunobiology, Vanderbilt University Medical Center, Nashville, TN USA; 8 Department of Pathology, Microbiology, and Immunology, Vanderbilt University Medical Center, Nashville, TN USA

**Keywords:** Cancer, Cancer, Endocrine system and metabolic diseases, Obesity

## Abstract

Medical therapeutics for weight loss are changing the landscape of obesity but impacts on obesity-associated cancer remain unclear. We report that in pre-clinical models with significant retatrutide (RETA, LY3437943)-induced weight loss, pancreatic cancer engraftment was reduced, tumor onset was delayed, and progression was attenuated resulting in a 14-fold reduction in tumor volume compared to only 4-fold reduction in single agonist semaglutide-treated mice. Despite weight re-gain after RETA withdrawal, the anti-tumor benefits of RETA persisted. Remarkably, RETA-induced protection extends to a lung cancer model with 50% reduced tumor engraftment, significantly delayed tumor onset, and mitigated tumor progression, with a 17-fold reduction in tumor volume compared to controls. RETA induced immune reprogramming systemically and in the tumor microenvironment with durable anti-tumor immunity evidenced by elevated circulating IL-6, increased antigen presenting cells, reduced immunosuppressive cells, and activation of pro-inflammatory pathways. In sum, our findings suggest that patients with RETA-mediated weight loss may also benefit from reduced cancer risk and improved outcomes.

## Introduction

Over 40% of the adult population in the U.S. is obese^1^. Obesity is associated with an increased risk of at least thirteen cancers and with negative cancer outcomes including increased metastasis, impaired response to therapy, elevated risk of recurrence, and an increase in cancer-related mortality^2–5^. Intentional weight loss has been reported to reduce obesity-associated cancer risk^6–9^. Lifestyle interventions, including exercise and nutritional approaches such as fasting or caloric restriction are often difficult to adopt, leading to low adherence and often weight cycling (loss and gain). However, weight loss after metabolic or bariatric surgery is highly effective in maintaining a reduced body weight for most patients^7,10^. Bariatric surgery is associated with both reduced cancer risk and mortality, demonstrating the potential for sustained weight loss to improve cancer outcomes^11,12^. Bariatric surgery has also been reported to reduce the risk of *non-obesity associated* cancers^13^. Thus, targeting obesity via supporting weight loss is an approach to prevent cancer onset and improve therapeutic outcomes^10,14^.

Recent advances in therapies to control diabetes have revolutionized obese patient care^15^. Medical weight loss intervention has been skyrocketing in recent years with many drugs on the market or in clinical trials, including incretin memetics or incretin receptor agonists such as Ozempic (semaglutide, “SEMA”) targeting glucagon-like peptide-1 (GLP-1) receptor (GLP1R) or dual agonist Mounjaro (tirzepatide) targeting GLP1R and glucose-dependent insulinotropic polypeptide (GIP) receptor (GIPR). Incretin agonism reduces appetite, promotes insulin release, and slows gastric transit, which together lead to profound weight loss with improvements in metabolism^16^. The advent of novel pharmacologic weight loss approaches is changing the landscape for comorbidities such as diabetes, cardiovascular, and renal dysfunction in patients with obesity. For the first time, this non-surgical approach is demonstrating sustained weight loss with few side effects. However, because incretin agonists are relatively new to the market for obesity, the impacts on cancer are just beginning to be understood. A retrospective study found that the use of GLP1R agonists was associated with lower risk for 10 of the 13 obesity-associated cancers in type 2 diabetes patients although the mechanisms remain unclear, suggesting a need for further preclinical and clinical investigation^17^.

To query whether weight loss by incretin agonism would impact cancer outcomes in pre-clinical models, we studied the impact of medical weight loss on two deadly cancers including pancreatic ductal adenocarcinoma (PDAC) and lung adenocarcinoma (LUAD) as either obesity-associated or obesity-independent cancers, respectively. PDAC is the third major cause of cancer-related deaths in the U.S. with only a 7.2% 5-year survival rate^2^. LUAD is the most prevalent form of lung cancer in the US and remains the leading cause of cancer-related deaths^18^. To examine impacts of incretin-mediated weight loss on the risk of PDAC and LUAD, we utilized the highly obesogenic C57BL/6J model with diet induced obesity (DIO). DIO mice were treated with SEMA or a novel therapeutic, retatrutide (“RETA,” LY-3437943) to induce weight loss. RETA is a triple hormone receptor peptide agonist targeting GLP1R, GIPR, and glucagon receptor (GCGR). In obese patients, SEMA induced a 16% reduction in weight while RETA demonstrated substantial weight loss up to 24%^19,20^. We compared the single agonist SEMA to the triple agonist RETA in DIO models and report that RETA potently improved cancer outcomes and revealed immune reprogramming as a potential underlying mechanism.

## Results

### RETA reduced body weight, improved metabolic parameters, and attenuated tumor outcomes in obesity-associated pancreatic ductal adenocarcinoma

**Fig. 1:**
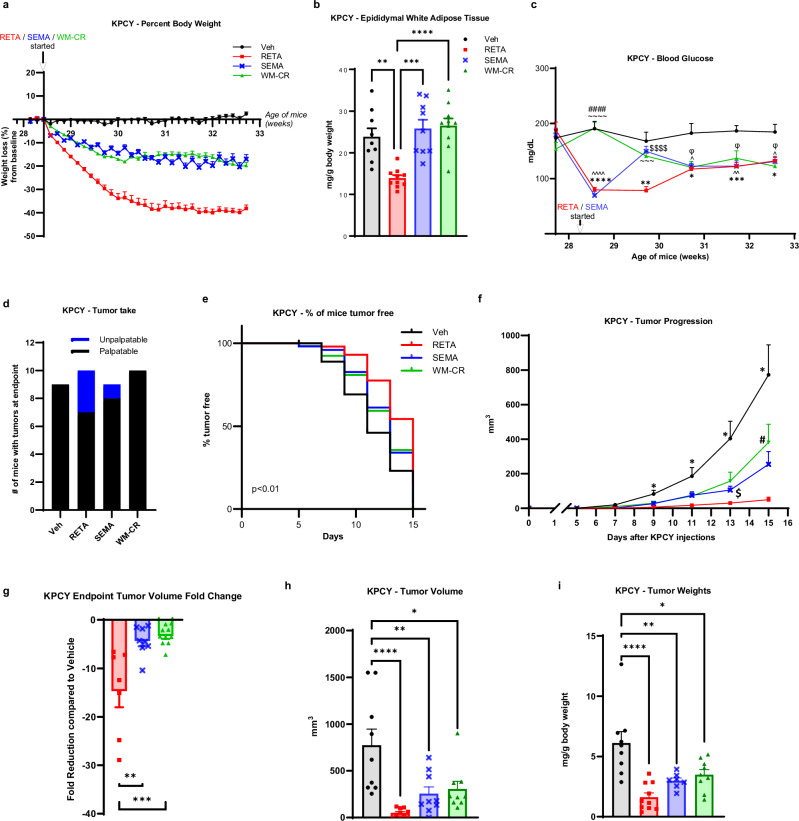
RETA or SEMA treatment reduces body weight, improves fasted blood glucose, and attenuates tumor outcomes in the KPCY model of obesity-associated PDAC. **a** Percent body weight changes from baseline are reported. **b**, Epididymal white adipose tissue weight was quantified at endpoint. **c** Fasted blood glucose was quantified at baseline and throughout the study at indicated timepoints until endpoint. **d** Tumor engraftment or “tumor take” is reported. **e** Survival curve is plotted as % of mice tumor-free for each group. **f** Tumor progression over 15 days after injecting KPCY cells is measured by volume recorded using digital caliper. **g** Fold change in tumor volume compared to Veh at endpoint are reported. **h** Endpoint tumor volumes, and (**i**) tumor weights (normalized to body weights) were quantified. Data are represented as mean ± SEM (*N* = 9 mice Veh, *N* = 10 mice RETA, *N* = 9 mice SEMA, *N* = 10 mice WM-CR). **b**, **g**, **h**, **i** Statistical significance was determined by one-way ANOVA with Tukey’s multiple comparison test (MCT) and is denoted as *P ≤ 0.05, **P ≤ 0.01, ***P ≤ 0.001, ****P ≤ 0.0001. **c**, **f** Statistical significance was determined by two-way ANOVA with Sidak’s MCT. **c** Statistical significance is denoted as *P ≤ 0.05, **P ≤ 0.01, ***P ≤ 0.001, ****P ≤ 0.0001, Veh versus RETA; ^*P* ≤ 0.05, ^^*P* ≤ 0.01, ^^^^*P* ≤ 0.0001 Veh versus SEMA, ^Φ^*P* ≤ 0.05 Veh versus WM-CR; ^$$$$^*P* ≤ 0.0001 RETA versus SEMA; ^~~~^*P* ≤ 0.001, ^~~~~^*P* ≤ 0.0001 RETA versus WM-CR; ^####^*P* ≤ 0.0001 SEMA versus WM-CR. **f** Statistical significance is denoted as *P ≤ 0.05 Veh versus RETA; ^$^*P* ≤ 0.05 RETA versus SEMA; ^#^*P* ≤ 0.05 RETA versus WM-CR.

First, impacts on PDAC demonstrated that both RETA and SEMA led to significant weight loss beginning one day after administration compared to vehicle (Veh, water) controls which remained weight stable (Fig. 1a, Supplementary Fig. 1a). All mice remained on high fat diet throughout the duration of the study. Weight loss in the RETA group was substantial and plateaued after 2 weeks of RETA intervention at 38% of initial weight at baseline, which was maintained until endpoint. In contrast, SEMA showed a gradual but “oscillatory” weight loss which fluctuated between 16 and 20% of initial weight which remained stable until endpoint (Fig. 1a). Although diet interventions in humans are difficult to maintain weight loss, mice respond to caloric restriction (CR) well; CR reduces cancer onset and progression in several models^21^. Therefore, weight loss through CR was induced to establish a weight matched control (WM-CR). Body weights were matched to the SEMA-treated mice to delineate the role of weight loss by CR as opposed to the role of GLP-1 agonism. Importantly, a WM-CR group for RETA could not be established because of the extreme weight loss observed with RETA which could not be implemented through CR alone. RETA and SEMA treatment was continued for the duration of the study. Results demonstrated that RETA and SEMA induced an immediate but transient reduction in food intake that rebounded after 2 weeks of treatment to control levels of intake quantified in the Veh group (Supplementary Fig. 1b). The food intake in WM-CR group was - by design - controlled to match weight loss in SEMA group (Supplementary Fig. 1b). Incretin agonism leads to delayed gastric emptying, which is one mechanism proposed to contribute to weight loss^22^. Cecal content weight at endpoint was quantified as an approximation for gastric emptying. Despite identical food intake and fasting at endpoint, while WM-CR did not alter cecal content mass compared to Veh controls, incretin agonism significantly elevated cecal content weight at endpoint. RETA demonstrated the most cecal content with 6.3-fold greater mass than Veh, and SEMA had half the mass found in RETA (Supplementary Fig. 1c). Future studies quantifying gastric emptying and gut transit are warranted^23^. Importantly, none of the mice in any of the groups displayed poor body condition score (*data not shown*).

Next, body composition and metabolic parameters were examined to determine improvement of metabolic dysfunction with incretin agonism. RETA led to remarkable improvements in body composition with a significant reduction in epididymal adipose mass quantified at endpoint as compared to Veh (Fig. 1b). Interestingly, despite significant weight loss observed in SEMA and WM-CR groups compared to Veh (Fig. 1a), no differences in epididymal fat mass were observed. The lack of loss in fat mass could be due to the “oscillatory” weight loss in SEMA group attributed to the alternate day dosing, as opposed to previous studies where SEMA was administered daily^24–26^. Similarly, CR in this study was designed to match body weights with SEMA group, while other CR approaches may differ^27,28^. Other factors may also impact differences such as age, source of mice, animal facility temperature, diet, and the microbiome which have demonstrated impacts on both obesity and cancer^29^. RETA treatment also resulted in striking improvements in systemic metabolism with a significant reduction in fasted blood glucose concentrations. RETA reduced blood glucose from ~ 190 mg/dL to approximately 80 mg/dL after 1 week, which steadily rose after 2 weeks of treatment over the remainder of the study to ~130 mg/dL while Veh controls maintained blood glucose ~ 180 mg/dL from baseline to endpoint (Fig. 1c). Despite a significant reduction in fasted blood glucose with SEMA treatment identical to RETA after 1 week, a striking rebound in blood glucose to almost baseline levels was observed by week 2 after treatment, which gradually reduced by endpoint. In contrast to incretin treatment, WM-CR showed a gradual decrease in fasted blood glucose throughout the study. Interestingly, all intervention groups showed similarly reduced fasted blood glucose concentrations at study endpoint that were significantly lower than Veh (Fig. 1c). Endpoint brown adipose tissue and liver weights were significantly reduced to similar levels by RETA, SEMA, and WM-CR compared to Veh; however, no differences in spleen weights were observed (Supplementary Fig. 1d–f).

Following robust weight loss with SEMA and even more dramatic weight loss with RETA, we next sought to determine impacts on PDAC. Kras^LSL-G12D/+^;Trp53^LSL-R172H/+^;Pdx1-Cre;Rosa26^YFP/YFP^ (KPCY) cancer cells^30^ were injected when RETA/SEMA weight loss plateaued after 2 weeks (Fig. 1a, Supplementary Fig. 1a). Quantification of tumor engraftment or “tumor take” demonstrated that RETA significantly reduced the number of resultant tumors after injection (Fig. 1d). While every mouse injected with cancer cells in the Veh and WM-CR groups had tumors engraft (*N* = 9/9, 100% Veh; *N* = 10/10, 100% WM-CR), in the RETA group, only 70% of the mice (*N* = 7/10) had tumors take while 30% mice rejected tumor cells at injection or failed to take. SEMA treatment showed higher tumor engraftment (*N* = 8/9, 88%) compared to RETA (Fig. 1d). Furthermore, thrice weekly palpation of KPCY injection sites revealed that RETA treatment significantly delayed tumor onset compared to Veh, SEMA and WM-CR (Fig. 1e). Tumors were allowed to progress for two weeks. While tumors in Veh group showed an exponential increase in volumes post day 7 after tumor cell injection, tumor volumes remained significantly blunted in RETA treated mice (Fig. 1f). SEMA treated mice showed tumor onset and progression at intermediate levels compared to RETA and Veh (Fig. 1e, f). WM-CR paralleled SEMA tumor onset and growth (Fig. 1e, f). Tumor volume at endpoint was most reduced by RETA, with a 14-fold reduction compared to Veh, while SEMA and WM-CR volumes did not differ with both at 3-fold reduction relative to Veh. (Fig. 1g, h). Endpoint tumor weights further demonstrated that compared to Veh, RETA exhibited the most pronounced effect among the treatment groups (Fig. 1i).

### RETA withdrawal reversed metabolic benefits, but tumor suppression remained persistent

**Fig. 2:**
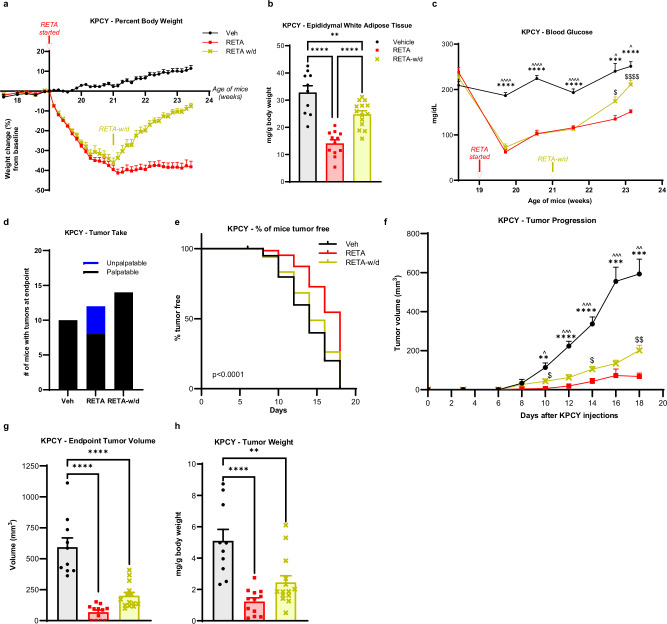
RETA withdrawal causes a reversal in body weight, adiposity, and fasted blood glucose, with only partial loss of anti-tumor effects in KPCY model of obesity-associated PDAC. **a** Percent body weight changes from baseline are reported. **b** Epididymal white adipose tissue weight was quantified at endpoint. **c** Fasted blood glucose was quantified at baseline and throughout the study at indicated timepoints until endpoint. **d** Tumor engraftment or “tumor take” is reported. **e** Survival curve is plotted as % of mice tumor-free for each group. **f** Tumor progression over 18 days after injecting KPCY cells was measured by volume recorded using digital caliper. **g** Endpoint tumor volumes, and (**h**), tumor weights (normalized to body weights) were quantified. Data are represented as mean ± SEM (N = 10 mice Veh, *N* = 12 mice RETA, *N* = 14 mice RETA-w/d). **b**, **g**, **h** Statistical significance was determined by one-way ANOVA with Tukey’s test and is denoted as **P ≤ 0.01, ****P ≤ 0.0001. **e** Log-rank (Mantel-Cox) test for survival curves (*P* < 0.0001). **c**, **f** Statistical significance was determined by two-way ANOVA with repeated measures using a mixed-effects model and Tukey’s multiple comparison test. **c** ***P ≤ 0.001, ****P ≤ 0.0001, Veh versus RETA; ^*P* ≤ 0.05, ^^^^*P* ≤ 0.0001, Veh versus RETA-w/d; $*P* ≤ 0.05, *P* ≤ 0.0001, RETA versus RETA-w/d. **f** **P ≤ 0.01, ***P ≤ 0.001, ****P ≤ 0.0001, Veh versus RETA; ^*P* ≤ 0.05, ^^*P* ≤ 0.01, ^^^*P* ≤ 0.001, Veh versus RETA-w/d; ^$^*P* ≤ 0.05, ^$$^*P* ≤ 0.01, RETA versus RETA-w/d.

Patients often do not tolerate the side effects of medical weight loss, including nausea or gut discomfort^31^, or may not have access to the prescriptions due to financial burdens or lack of availability. We wondered if weight rebound would reverse the profound protective effects of RETA to reduce PDAC onset and burden. Therefore, we examined the effect of RETA withdrawal (RETA-w/d) on metabolic parameters and PDAC (Supplementary Fig. 1a). A second group of mice was examined for RETA-induced weight loss and RETA-w/d compared to Veh controls. Similar to the first study, RETA intervention led to a transient reduction in food intake and a 40% reduction in body weight that stabilized after two weeks for the duration of the study (Fig. 2a, Supplementary Fig. 2a). Mice subjected to RETA-w/d displayed an immediate weight regain almost to baseline body weights (Fig. 2a). Food consumption after RETA withdrawal significantly increased and, in fact, surpassed that of Veh for 2 weeks before returning to baseline levels of intake at study endpoint, not significantly different from Veh or RETA intake (Supplementary Fig. 2a). Similarly to the first study, RETA delayed gastric emptying, whereas RETA-w/d completely reversed cecal retention and returned to reduced cecal content weights comparable to Veh (Supplementary Fig. 2b). The fact that the cecal contents of RETA-w/d mice are not different than Veh controls demonstrates that RETA withdrawal was effective as the half-life in mice is 21 h^32^.

RETA again led to significant improvements in body composition that were partially retained after RETA withdrawal despite the dramatic weight rebound in the RETA-w/d group. A significant reduction in epididymal fat mass was quantified at endpoint after RETA and reflected by an almost ablated concentration of circulating leptin, whereas after RETA withdrawal, white adipose mass and leptin concentrations were partially reversed compared to Veh (Fig. 2b, Supplementary Fig. 2c). Similarly, endpoint brown adipose and liver weights were reduced by RETA and regained after RETA-w/d to Veh weights (Supplementary Figs. 2d, e); however, spleen weights were increased with RETA treatment and restored with RETA withdrawal (Supplementary Fig. 2f).

Next, metabolic parameters after incretin agonist withdrawal were examined to determine the extent of improvement of metabolic dysfunction^33^. Veh treated mice displayed glucose concentrations of >200 mg/dL throughout the study (Fig. 2c). The striking improvements in systemic metabolism with RETA treatment were partially reversed after RETA-w/d. Although RETA induced a significant reduction in fasted blood glucose concentrations after 1 week, which steadily rose over time but remained significantly reduced compared to Veh, RETA-w/d caused a sharp increase in fasting blood glucose (Fig. 2c). Compared to Veh, blood glucose levels were 40% lower in RETA and 16% lower after RETA-w/d at endpoint (Fig. 2c). Next, to examine functional responses to glucose after RETA or RETA-w/d, oral glucose tolerance tests (OGTT) were performed. RETA improved glucose tolerance compared to baseline tolerance tests at the start of the study, as well as compared to Veh at study midpoint (Supplementary Figs. 3a, b). Interestingly, by endpoint, all three groups displayed elevated glucose concentrations to the same extent 15 mins after glucose administration despite significantly different fasted concentrations at time 0 (Supplementary Fig. 3c). Despite glucose concentrations peaking at approximately 400 mg/dL during the OGTT, both RETA and RETA-w/d groups disposed of glucose effectively by the end of the study compared to DIO mice treated with Veh, which displayed glucose intolerance (Supplementary Fig. 3c). Importantly, RETA also significantly reduced fasting plasma insulin concentrations compared to Veh at endpoint, which were only partially reversed after RETA-w/d (Supplementary Fig. 3d). C-peptide, a peptide released from pro-insulin to generate mature insulin, is a proxy measure for insulin release. Likewise, C-peptide concentrations paralleled those of insulin with a significant reduction in the RETA group, which was only partially reversed to Veh concentrations after RETA-w/d (Supplementary Fig. 3e). Resistin, an adipokine which may play a role in insulin resistance^34^, was also significantly downregulated with RETA-induced weight loss but was restored to Veh concentrations after RETA-w/d (Supplementary Fig. 3f). A calculated estimate of insulin resistance using the homeostatic model assessment for insulin resistance (HOMA-IR) demonstrated a significant reduction in the HOMA-IR score to almost undetectable levels with RETA treatment, an approximation suggesting a loss of insulin resistance (Supplementary Fig. 3g). With RETA withdrawal, endpoint analysis revealed that HOMA-IR was restored to just 40% of the Veh group (Supplementary Fig. 3g). Furthermore, a significantly higher quantitative insulin-sensitivity check index (QUICKI) was observed for RETA-treated mice compared to vehicle and RETA-w/d groups, suggesting improved insulin sensitivity with RETA (Supplementary Fig. 3h).

Inspired by the remarkable findings in PDAC with RETA treatment, we next sought to determine the effect of RETA withdrawal on PDAC tumor onset and progression. Tumor take after KPCY injection was significantly reduced by RETA with only 66% tumor engraftment (*N* = 8/12), as compared to 100% in Veh (*N* = 10/10) (Fig. 2d), similar to Fig. 1d. Of note, the protection from RETA to reject tumors was completely lost after RETA withdrawal with all tumors engrafting (*N* = 14/14, 100%, Fig. 2d). RETA again led to significantly delayed tumor onset and blunted tumor growth over the course of the study (Fig. 2e, f), as in our first study (Fig. 1e, f). Likewise, tumor volume and weights were significantly reduced at endpoint by RETA compared to Veh controls (Fig. 2g, h). Tumor onset and growth dynamics after RETA withdrawal showed partial protection in tumor onset and persistent protection in tumor progression with significantly reduced endpoint tumor volume and weight compared to Veh. Moreover, tumor progression after RETA withdrawal was also significantly different relative to RETA-treated mice (Fig. 2e–h). Lastly, we examined classic circulating markers of obesity-associated inflammation that are associated with cancer^35^. Despite the striking differences observed in metabolic measures at endpoint, no changes were seen in circulating inflammatory markers TNF-α and MCP-1/CCL2 in RETA or RETA-w/d groups compared to Veh (Supplementary Figs. 3 i, j). However, RETA moderately increased IL-6 concentrations, whereas withdrawal caused significantly elevated plasma IL-6 concentrations compared to Veh (Supplementary Fig. 3k). These findings suggest that RETA leads to persistent changes that impair tumor progression even after discontinuing treatment.

**Fig. 3:**
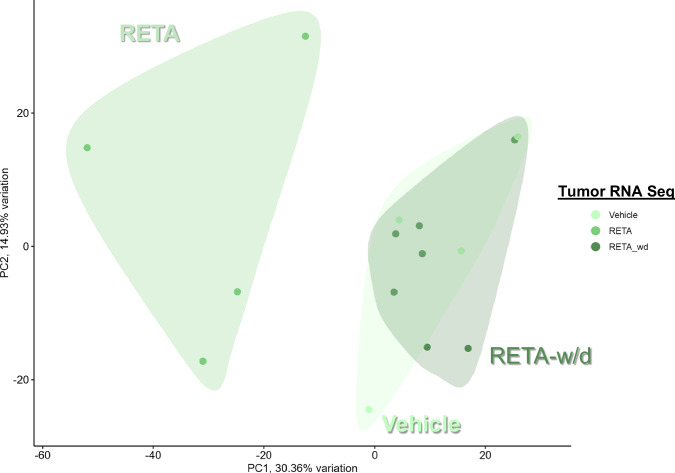
RETA treatment significantly alters gene expression in KPCY tumors which is restored with RETA-w/d. Principal component analysis (PCA) plots of gene expression analysis using RNAseq data is shown.

**Fig. 4:**
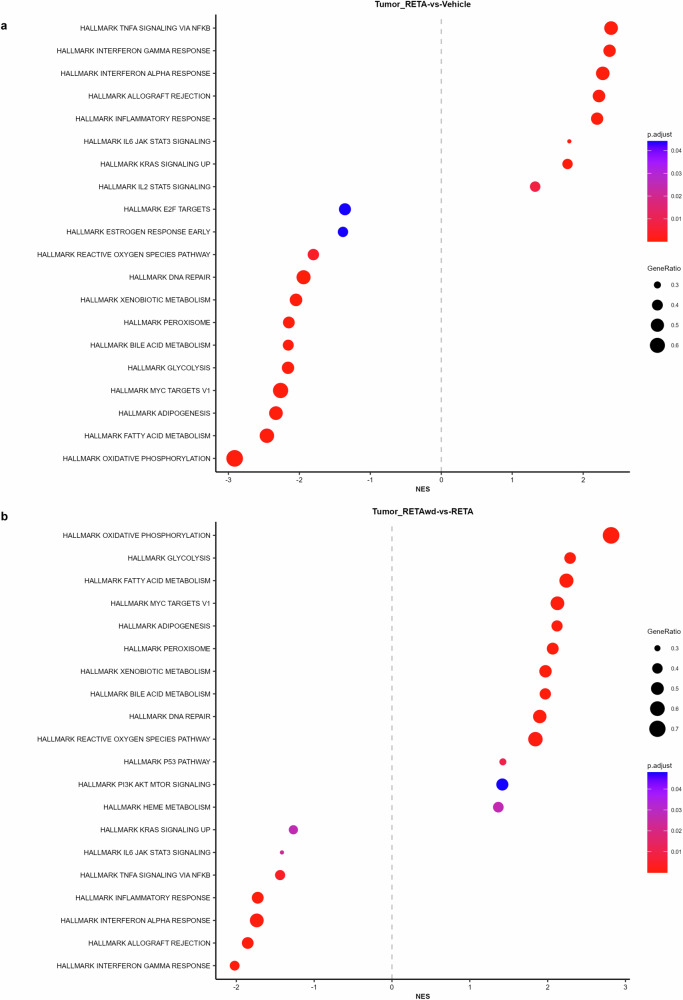
RETA treatment significantly modulates hallmark pathways involved in improved tumor outcomes, which is reversed with RETA-w/d in the KPCY model. Modulation of various immunometabolic Hallmark pathways influencing tumor outcomes are reported for (**a**), RETA versus Veh and (**b**), RETA-w/d versus RETA.

Therefore, we evaluated the effects of RETA and RETA-w/d on the tumor microenvironment to determine if there is a persistent effect of RETA. Principal Component Analysis (PCA) of KPCY tumor RNA-seq transcriptomic data revealed significantly different and highly distinct clustering of RETA treated mice compared to Veh controls (Fig. 3). Interestingly, tumors from the RETA-w/d group completely resembled the Veh group (Fig. 3). Gene Set Enrichment Analysis (GSEA) uncovered unique impacts of RETA on key biological processes. Compared to Veh, RETA treatment significantly enriched the expression of genes associated with several Hallmark pathways such as TNFA signaling via NFκB, interferon gamma and alpha responses, and inflammatory response which are associated with enhanced anti-tumor immunity (Fig. 4a). Additionally, Hallmark pathways such as IL2 STAT5 signaling and allograft rejection were upregulated, reflecting the heightened immune activation in tumors which could foster a hostile environment for tumor growth. In contrast, downregulation of hallmark E2F targets and MYC targets V1 with RETA hints at impaired cancer cell proliferation and tumor suppression (Fig. 4a). Lastly, significant downregulation of metabolic pathways such as bile acid metabolism, glycolysis, fatty acid metabolism, and oxidative phosphorylation could contribute to tumor suppression by regulating anti-tumor immunity and tumor growth (Fig. 4a).

Interestingly, RETA withdrawal reversed the regulation of almost all of the Hallmark pathways regulated by RETA. Hallmark pathways such as oxidative phosphorylation, glycolysis, fatty acid metabolism, MYC targets V1, adipogenesis, and bile acid metabolism were significantly upregulated in tumors from mice subjected to RETA-w/d (Fig. 4b). In contrast, Hallmark pathways such as TNFA signaling via NFκB, interferon gamma and alpha responses, inflammatory response, and allograft rejection were downregulated (Fig. 4b).

### RETA treatment improved metabolic factors, boosted anti-tumor immunity, and potently blunted tumor progression in non-obesity associated lung adenocarcinoma

**Fig. 5:**
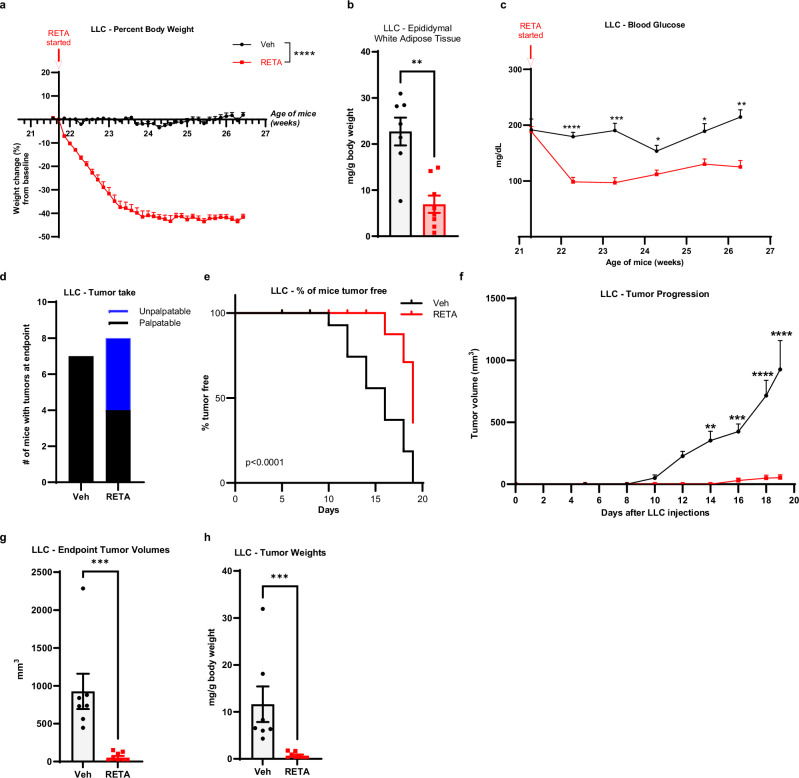
RETA treatment reduces body weights and adiposity, improves fasted blood glucose, and attenuates tumor outcomes in LLC model. **a** Percent body weight changes from baseline are reported. **b** Epididymal white adipose tissue were weighed at endpoint and normalized to total body weight. **c** Fasted blood glucose was quantified at baseline and throughout the study at indicated timepoints until endpoint. **d** Tumor engraftment or “tumor take” is reported. **e** Survival curve plotted as % of mice tumor-free for each group. **f** Tumor progression over 19 days after injecting LLC cells measured by volume recorded using digital caliper. **g** Endpoint tumor volumes, and (**h**), tumor weights (normalized to body weights) were quantified. Data are represented as mean ± SEM (*N* = 7 mice Veh, *N* = 8 mice RETA). **a** Statistical significance was determined by Two way ANOVA and is denoted as ****P ≤ 0.0001. **b**, **g**, **h** Statistical significance was determined by Student’s *t*-test with Mann-Whitney test and is denoted as **P ≤ 0.01, ***P ≤ 0.001. **c**, **f** Statistical significance was determined by two-way ANOVA with repeated measures using a mixed-effects model and Sidak’s multiple comparison test. *P ≤ 0.05; **P ≤ 0.01; ***P ≤ 0.001; ****P ≤ 0.0001. **e** Log-rank (Mantel-Cox) test for survival curves (*P* < 0.0001).

Based on the dramatic findings in PDAC, an obesity-associated cancer, we were interested in whether RETA could also be effective in a non-obesity associated cancer. Thus, we next studied RETA treatment in a Lewis lung carcinoma (LLC) mouse model of LUAD using an identical study design to KPCY above in the same strain of mice, C57BL/6 J with DIO (Supplementary Fig. 1a). In line with the KPCY model, RETA effectively reduced body weights within the first two weeks, plateauing at 41% loss of baseline body weight (Fig. 5a). Significant reductions in epididymal white adipose mass and circulating leptin were found in RETA compared to Veh (Fig. 5b, Supplementary Fig. 4a). Like above, RETA significantly reduced gastric emptying in LLC model with RETA cecal content greatly elevated compared to Veh (Supplementary Fig. 4b). Similarly, endpoint brown adipose tissue and liver weights were significantly reduced with RETA, however there were no differences in spleen weight (Supplementary Fig. 4c–e). RETA also improved systemic metabolism in LLC model with a significant reduction in fasted blood glucose concentrations 1 week after starting the treatment continuing until endpoint where glucose concentrations were 41% lower compared to Veh (Fig. 5c), which was similar to findings in the KPCY studies above. Compared to Veh, RETA also showed significantly lower concentrations of plasma insulin at endpoint and reduced HOMA-IR score, whereas the QUICKI score was significantly increased with RETA (Supplementary Fig. 4f–h). Taken together, in this LLC model, RETA induced identical physiological improvements compared to the KPCY cohorts, demonstrating highly reproducible impacts on metabolic outcomes.

Once improved metabolic parameters were established after RETA treatment, we assessed the impact of RETA on LUAD. After two weeks of RETA treatment, LLC cells were injected when weight loss plateaued as in above studies (Supplementary Fig. 1a). Importantly, tumor take was remarkably reduced with RETA (*N* = 4/8, 50%) compared to Veh (*N* = 7/7, 100%) (Fig. 5d), more than observed with RETA treatment in the KPCY model. RETA treatment dramatically delayed LLC tumor onset, with unpalpable tumors in the RETA treated group until day 16, compared to onset observed in Veh treated mice where tumors were palpated starting on day 10 (Fig. 5e). RETA also blunted tumor progression with significantly reduced tumor volumes and weights at endpoint compared to Veh (Fig. 5f–h). Lastly, circulating cytokines and chemokines were measured with minor non-significant elevations in plasma concentrations of TNF-α and MCP-1 after RETA treatment (Supplementary Fig. 4i, j). However, IL-6 was significantly 4.6-fold elevated with RETA treatment in the LLC model (Supplementary Fig. 4k).

**Fig. 6:**
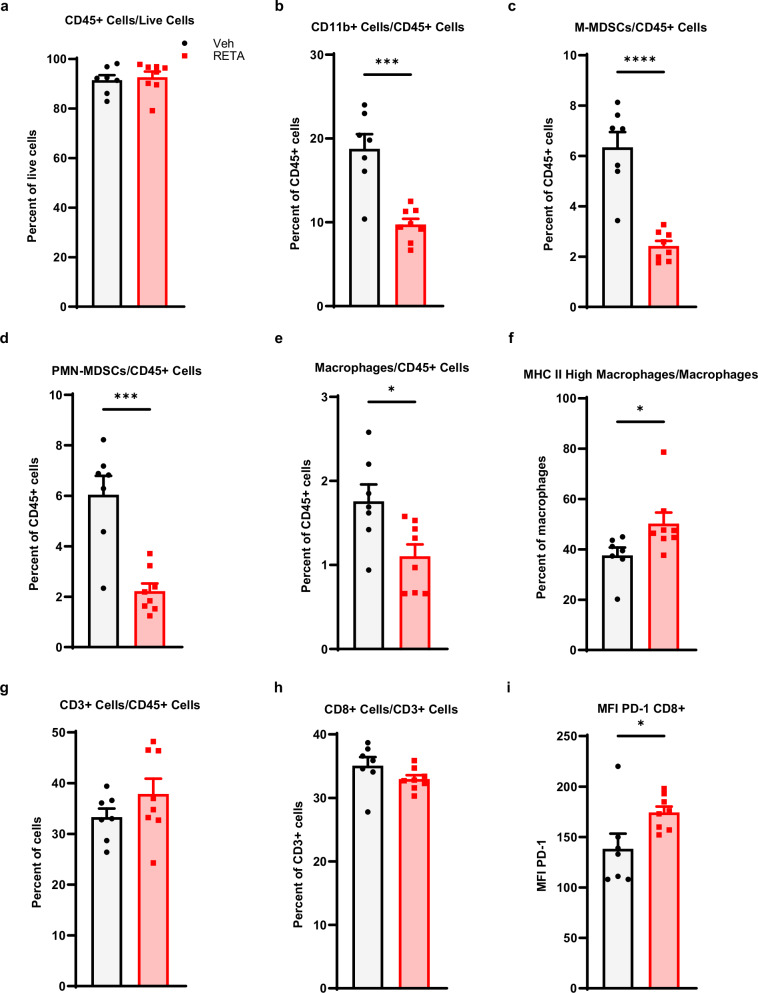
RETA significantly improves antigen presentation and reduces immunosuppression in the LLC model. Frequencies of (**a**), CD45+ cells/live cells (**b**), CD11b+ cells/CD45+ cells, (**c**), M-MDSCs/CD45+ cells, (**d**), PMN-MDSCs/CD45+ cells, (**e**), macrophages/CD45+ cells, **f**, MHC II high macrophages/macrophages, **g** CD3+ cells/CD45+ cells, and (**h**), CD8+ cells/CD3+ cells are shown. **i** The mean fluorescence intensity for PD-1 is shown for CD8+ cells. Data is represented as mean ± SEM (*N* = 7 mice Veh, *N* = 8 mice RETA).

Last, to assess the role of the anti-tumor immunity to RETA-mediated improved LLC tumor outcomes, we examined systemic quantities of key innate and adaptive immune cells. A significant reduction in CD11b+ cells and macrophages was observed with RETA when quantified out of CD45, which did not differ between intervention and Veh (Fig. 6a, b). Intriguingly, RETA significantly decreased monocytic myeloid derived suppressor cells (M-MDSCs) and granulocytic or polymorphonuclear MDSCs (PMN-MDSCs) and enriched MHC II high macrophages (Fig. 6c–f), suggesting reduced immunosuppression and increased antigen presentation with RETA, which aligns with reduced tumor burden. Lastly, while no significant changes were observed in CD3+ or CD8+ cells, RETA significantly increased PD-1 mean fluorescent intensity (MFI) in CD8+ T cells compared to Veh, suggesting elevated activation of cytotoxic T cells (Fig. 6g–i).

## Discussion

RETA dramatically reduced tumor onset and burden in obesity-associated PDAC and non-obesity-associated LUAD. Several potential mechanisms were examined from metabolic to endocrine to immune mediators which could underlie the significant reductions in tumor burden. RETA induced a significant yet transient decrease in food intake and substantial weight loss across three cohorts of DIO mice with consistent improvements in glucose tolerance and insulin resistance compared to Veh controls. Compared to obese mice treated with Veh, KPCY and LLC tumors in mice treated with RETA failed to engraft 33-50% of the time and did not grow appreciably, which suggests that RETA may impact both cancer risk and progression.

Our study examined two incretin mimetics, with SEMA currently FDA-approved for both diabetes and obesity while RETA is still in clinical trials. The comparison of SEMA to RETA is important because SEMA is a single agonist targeting only GLP1R while RETA is a triple agonist targeting GLP1R plus GIPR and GCGR. We also examined an important intervention to control for weight loss through matching the mass of the SEMA treated group by restricting dietary intake. We report that while SEMA led to substantial weight loss, reduced tumor engraftment, blunted progression, and reduced tumor burden compared to Veh, RETA demonstrated superior effectiveness with remarkable and sustained weight loss, profound reductions in tumor engraftment, and significantly reduced tumor burden compared to SEMA, WM-CR, and Veh. Notably, WM-CR mice exhibited identical tumor onset and similar progression to SEMA, which supports that weight loss alone may be beneficial independent of GLP1R agonism. Blood glucose, food intake, and major tissues including brown adipose, liver, and spleen weights were similar between RETA and SEMA, yet RETA uniquely reduced adiposity and delayed tumor latency suggesting that targeting a single receptor GLP1R was effective for many physiologic and metabolic benefits, but the additional agonism of GIPR and GPCR by RETA uniquely impacted adiposity, tumor onset, and tumor progression. Importantly, elevated fasting glucose and elevated insulin driven by insulin resistance has been associated with pancreatic cancer^36,37^. Thus, the fact that the observed benefits of RETA against PDAC could be indirect by inducing weight loss and improving metabolic parameters - such as blood glucose and insulin - cannot be excluded and need further investigation.

A limitation of this study is that the weight loss in the RETA group could not be matched simply by restricting food intake due to the many pathways targeted by RETA leading to, for example, significantly elevated cecal content indicative of reduced gastric motility. To address this limitation, we discontinued RETA treatment, which is important because many patients do not tolerate or cannot afford weight loss medications long term. Halting RETA therapy at study midpoint induced a rebound of body weight and elevated food intake, which resulted in restoration of cecal content, adipose and liver mass, and an altogether accelerated weight gain. Our findings provide important evidence supporting clinical reports of weight gain following incretin agonist discontinuation in patients^38^. With regard to tumor outcomes, RETA withdrawal surprisingly led to persistently limited tumor progression despite weight regain and elevated food intake which even transiently surpassed RETA and Veh group’s intake. Examination of circulating inflammatory markers showed that RETA moderately increased IL-6 concentrations, whereas withdrawal significantly elevated plasma IL-6 concentrations compared to Veh. IL-6 has a complicated and often contradictory role based on its cell or tissue of origin, concentration, and signaling pathways spanning from pro- to anti-inflammatory roles especially with regard to obesity and inflammation^39–43^. In cancer, IL-6 can either be pro- or anti-inflammatory depending on the context and can thus demonstrate pro- or anti-tumor effects^44^. Taken together, RETA led to remarkable reductions in tumor progression that persisted even when the intervention was withdrawn and weight rebounded along with elevated adipokines and cytokines associated with cancer, namely leptin and IL-6. Despite a half-life of RETA of 21 hours in mice, a protective effect on tumors was observed after RETA withdrawal evident from a reduced progression compared to Veh. The sustained protection observed after RETA withdrawal indicates its anti-tumor potential, even if weight loss is not maintained. This warrants further investigation in future prospective patient cohorts.

Transcriptomic analysis has provided potential insight into the mechanistic role RETA could play in the substantially blunted tumor progression through both immune- and metabolism-mediated mechanisms which may be reversed with RETA-w/d. Importantly, although RETA-w/d restored the tumor transcriptome close to that of Veh, the tumor progression remained relatively blunted, which could be due to factors external to the tumor including systemic metabolic effects, such as insulin concentration, which were only partially restored after RETA-w/d, as discussed above. Thus, a major limitation of this study is that reported, or as yet unexplored, systemic metabolic mediators may impact tumor outcomes, therefore the findings highlight the need for additional research to explore the precise mechanisms through which GLP-receptor agonists improve tumor outcomes.

Taken together, analysis of endpoint physiologic and metabolic parameters demonstrated that RETA induced potent improvements in systemic glucose tolerance and insulin sensitivity across several studies, as is well established^19^. However, RETA appears to provide mainly transient protective metabolic benefits due to reversal or partial reversal of several critical parameters including adipose tissue mass, blood glucose, insulin, resistin, and associated approximations of insulin sensitivity or resistance. Importantly, despite lung cancer not being identified as an obesity-associated cancer type, RETA also demonstrated dramatic improvements in the LLC model of LUAD with significant improvements in metabolic parameters. RETA dramatically blunted LUAD onset, progression, and burden with significant changes in systemic anti-tumor immune cytokine IL-6, antigen presenting macrophages, and activated CD8+ T cells, with significantly reduced immunosuppressive cells, which together support that RETA leads to a broad and durable promotion of anti-tumor immunity.

Clinically, GLP-1 agonists appear to have a broadly beneficial impact on cancer prevention as supported by this report. In a large retrospective cohort study including over 1.6 million patients with Type 2 Diabetes, with no prior diagnosis of obesity associated cancers, a significantly reduced risk in 10 out of 13 obesity associated cancers was reported with the use of GLP-1 agonists, including esophageal, colorectal, endometrial, gallbladder, kidney, liver, ovarian, pancreatic cancer, meningioma, and multiple myeloma^17^. Although the detrimental effect of GLP-1 agonists have remained unclear with suggestions of increased risk of some cancers, recent reports in fact suggest a reduced risk of colorectal cancer (CRC). On the contrary, reports on the association of GLP-1 agonists with the risk of thyroid cancer remain inconsistent. A reduced risk of CRC was reported in a retrospective study of over 1 million individuals with type 2 diabetes (T2D)^45^. Importantly, the association was more pronounced in patients with obesity/overweight, indicating a potential protective effect against CRC that may be partly driven by weight loss, as well as other mechanisms unrelated to weight loss. Genetic alteration, expression analysis, and in vitro evidence further support that reduced GLP-1 signaling is associated with worse survival across CRC and other cancers. Interestingly, elevated GLP-1 signaling is associated with greater immune infiltration and better response to immunotherapy^46^. With regard to thyroid cancer, the current understanding in the field is less clear. A nested case-control study analyzed data from the French national health care insurance system (SNDS) on individuals with T2D treated with second-line anti-diabetes drugs from 2006 to 2018, reported an increased risk of all thyroid cancer and medullary thyroid cancer with use of GLP-1 agonists after 1–3 years of treatment^47^. Furthermore, in a meta-analysis study, a moderate increase in the relative risk and a slight increase in absolute risk of thyroid cancer with the use of GLP-1 agonists was reported^48^. On the other hand, other studies have reported no association between the use of GLP-1 agonists and the risk of thyroid cancer^49,50^. In sum, GLP-1 agonists associate with a reduced risk in CRC and a modest relative increase in thyroid cancer risk; however, further study is warranted to conclude whether and to what extent GLP-1 agonists elevate thyroid cancer risk. Findings may suggest that in individuals without specific risk factors for thyroid cancer, the overall benefits of GLP-1 agonists may surpass any potential harm.

Incretin agonists are groundbreaking advances in medicine, enabling for the first time the study of non-surgical interventions for effective and sustained weight loss, which is presenting many exciting avenues for future investigation. Bariatric surgery-mediated weight loss is associated with both reduced cancer risk and mortality^11^, with improved therapeutic effectiveness, although the mechanisms remain unclear. It is uncertain if patients taking various incretin agonists will be revealed to have the same impressive benefits. Our work using several complementary pre-clinical approaches has demonstrated that RETA potently improved metabolic dysfunction associated with obesity and demonstrated effective anti-tumor potential against obesity-associated and non-obesity-associated cancer in mice. RETA may be effective in cancer prevention or interception for those at elevated risk of PDAC and LUAD, or other cancers. In summary, findings presented herein have profound implications for patients currently considering or taking incretin mimetics for diabetes or weight loss on cancer outcomes.

## Methods

### Reagents

Unless otherwise specified, all reagents were purchased from Sigma-Aldrich (St. Louis, MO). Retatrutide (RETA) (LY3437943, Cat. No. HY-P3506, MedChemExpress, Monmouth, NJ), Semaglutide (SEMA, Cat. No. HY-114118, MedChemExpress, Monmouth, NJ) and dextrose (CAS No. 50-99-7, Fisher Scientific, Waltham, MA) were purchased from Fisher Scientific Company, Waltham, MA. PCR grade water was purchased from Alpha Teknova (Product number W3330, CAS No. 7732-18-5, Alpha Teknova, Inc., Hollister, CA). Flow cytometry antibodies (Table 1), compensation beads, and reagents were from Tonbo Biosciences, Inc. (San Diego, CA) and Biolegend (San Diego, CA).

**Table 1:** Antibodies for flow cytometric analysis of spleens with myeloid and T cell panels.

Antibody	Source	Identifiers	Dilutions
*Myeloid Panel*			
Anti-Human/Mouse CD11b redFluor 710	Tonbo	80-0112-U025	1:20
APC anti-mouse Ly-6C	BioLegend	128015	1:40
Anti-Mouse F4/80 Antigen PE	Tonbo	50-4801-U025	1:40
Anti-Mouse Ly-6G PerCP-Cyanine5.5	Tonbo	65-1276-U025	1:40
PE/Cyanine7 anti-mouse I-A/I-E (MHC class II)	BioLegend	107630	1:40
FITC anti-mouse CD45	BioLegend	103108	1:40
*T Cell Panel*			
Anti-Mouse CD8a FITC	Tonbo	35-0081-U025	1:10
Brilliant Violet 605 anti-mouse CD4	BioLegend	100548	1:10
Brilliant Violet 785 anti-mouse CD3ε	BioLegend	100355	1:04
Brilliant Violet 421 anti-mouse CD279 (PD-1)	BioLegend	109121	1:10
PerCP/Cyanine5.5 anti-mouse CD45	BioLegend	103132	1:20

### Animal experiments

The protocol (IACUC #24-0504.0) used for animal studies was approved by the University of Tennessee Health Science Center’s (UTHSC) Institutional Animal Care and Use Committee (IACUC) under Animal Welfare Assurance Number A3325-01 and in accordance with the National Institutes of Health Guide for the Care and Use of Laboratory Animals. Diet-induced obese (DIO) C57BL/6 J male mice were purchased from Jackson Laboratory (#380050, Bar Harbor, ME) at 16 weeks of age. Upon receipt, mice were housed in groups of five and acclimated for 3 weeks. The mice were maintained on the same diet used to induce DIO by Jackson Lab, 60 kcal% from high-fat diet (D12492i, Research Diets Inc., New Brunswick, NJ, USA) with *ad libitum* access and sterile water was provided from the Lixit System. A 12 h light:dark cycle and temperature at 24 ± 2 °C was maintained. The health of the mice was monitored using body condition scoring (BCS) to observe any signs of weakness or inactivity.

### RETA and SEMA administration

The study design is shown in Supplementary Fig. 1a. Mice were randomized to intervention at the start of the study at the cage level. Mice were assigned to respective study groups to comprehensively evaluate multiple medical weight loss approaches with various controls: 1) vehicle (Veh, sterile water) as control, 2) SEMA to induce weight loss using semaglutide, 2) WM-CR to induce weight loss by caloric restriction to match weight loss in SEMA, 3) continuous RETA to induce weight loss, or 4) short-term RETA to induce weight loss then regain, termed “RETA withdrawal” (RETA-w/d). Mice were subcutaneously (SQ) administered either Veh, 30 nmol/kg RETA^32^, or 30 nmol/kg SEMA every other day before the initiation of the dark cycle at 5 pm. In the RETA-w/d group, 14 days after RETA initiation, RETA administration was stopped, and this subset of mice was switched to SQ Veh injections. The body weight of mice and food intake were recorded daily.

### Cell culture

KPCY 2838 mouse pancreatic cancer cell line was acquired from Kerafast^®^ (Shirley, MA). KPCY cells were cultured in DMEM (Cat. No. 11965092, Gibco, Waltham, MA) supplemented with 1% penicillin-streptomycin (Cat. No. SV30010, HyClone™, Cytiva, Marlborough, MA), 1% GlutaMAX (Cat. No. 35050061, Gibco, Waltham, MA), and 10% FBS (Cat. No. 26140-087 Gibco, Waltham, MA) per instructions from Kerafast^®^. The cells were cultured in 150 mm^2^ culture dishes containing 25 mL media under aseptic conditions in a CO_2_ incubator at 37 °C. The cells were passaged after reaching a sub-confluent stage. LLC cell line was a gift from J.A. Carson (UTHSC, Memphis, TN) and was cultured in DMEM as described previously^51^. Cell lines were confirmed negative for mycoplasma.

### Tumor models and endpoint

DIO mice were injected SQ with either 2 × 10^5^ KPCY cells or 10^6^ LLC cells in 100 µL sterile PBS in the right flank. Mice were maintained on Veh, SEMA, WM-CR, or RETA (Supplementary Fig. 1a). Tumor implantation (tumor take) was recorded. The progression of the tumors was monitored by palpating every 3 days after injecting the cells until tumors were palpable, after which the tumors were measured by digital caliper every other day. Tumor size was monitored by measuring the length and width, and endpoint was 2.5 weeks after cell injection. Tumor volume was calculated using the formula: volume = (length) × (width)^2^/2^52^. At the endpoint, mice were euthanized using isoflurane. Blood was collected via cardiac puncture into EDTA-coated syringes. Plasma was separated from other blood components by centrifugation at 1200 × g for 45 min at 10 °C. Tumors were excised and tumor mass was recorded. Cecal content was collected and weighed. Epididymal white adipose tissue (WAT), brown adipose tissue (BAT), liver, and spleen were collected and weighed. Samples were snap-frozen in liquid nitrogen and stored at ^−^80 °C until further analysis.

### Oral glucose tolerance test (OGTT)

For the KPCY model, oral glucose tolerance tests (OGTT) were performed to evaluate glucose tolerance^53,54^. OGTTs were performed two days before beginning the RETA treatment (baseline OGTT), 12 days after beginning RETA/Veh treatment (midpoint OGTT) before KPCY cell injections, and 2 days before endpoint (endpoint OGTT), (Supplementary Fig 1a). Briefly, mice were fasted for five hours in fresh cages with *ad libitum* access to drinking water. Blood glucose levels were measured using tail vein prick using a lancet and a Glucometer (Contour^TM^ Next EZ, Ascensia Diabetes Care Holdings AG, Basel, CH). Blood glucose readings for 0 min time point were recorded, then 2 g/kg dextrose was administered using a 20% glucose solution prepared in sterile distilled water by oral gavage. Blood glucose was measured at 15, 30, 60, 90, and 120 min. Bleeding was stopped by applying Kwik Stop^®^ Styptic powder (Miracle Care Products, Dayton, OH) using sterile cotton swabs.

### Cytokine profiling using immunology multiplex assay

MCP-1/CCL2, IL-6, and TNF-alpha (TNFα) were measured in fasted plasma collected at endpoint using the Milliplex MAP Mouse Metabolic Hormone Magnetic Bead Panel in the Luminex MAGPIX system (EMD Millipore, Billerica, MA).

### Bulk RNAseq analysis

Reads from RNAseq data were processed using the nf-core/RNAseq v3.17.0 pipeline and the GRCm39 genome assembly^55^. Aligned transcript counts were imported into R v4.2.2 using Tximport v1.32.0^56^. Low capture genes with less than ten total counts summed across all samples were removed. Differential expression was performed using DESeq2^57^. Gene set enrichment analysis was performed using fgsea v1.30.0 using mouse-ortholog Hallmark genesets from MsigDB loaded with msigdbr v7.5.1^58,59^. Data is deposited in NCBI GEO (# GSE288998).

### Flow cytometry

Excised spleens were crushed in RPMI following established protocols^52,60^. Digested tissue was filtered through 70 µm strainer to obtain a single cell suspension. Antibodies used (Table 1) were titrated and the separation index was calculated using FlowJo version 10 software for every study. Following red blood cell lysis (BioLegend), viability was determined by staining with Ghost dye (Tonbo Biosciences, Inc.) followed by FcR-blocking (Tonbo Biosciences, Inc.). Cells were stained with fluorescently labeled antibodies and fixed in Perm/fix buffer (Tonbo Biosciences Inc.). Stained cells were analyzed using a Bio-Rad ZE5 flow cytometer at the UTHSC Flow Cytometry and Cell Sorting Core. A minimum number of 1000 events were considered for analysis. Fluorescence minus one (FMO) stained cells and single color Ultracomp Beads (Invitrogen, Carlsbad, CA) were used as negative and positive controls, respectively. Data were analyzed using FlowJo version 10 software with FlowAI plug in clean up. Gating schemata were drawn in FlowJo version 10 software (Supplementary Fig. 5).

### Statistical methods

Statistical analysis was conducted using One-way or Two-way ANOVA followed by Tukey’s or Sidak’s multiple comparison test or using Student’s *t*-test as specified in figure legends. Data are presented as mean ± SEM. P value less than 0.05 was considered statistically significant. Data and statistical analysis were performed using GraphPad Prism 10.4.0 (Graphpad Software, Inc., La Jolla CA). Animals were assigned to study groups based on their starting body weights to ensure equal unbiased distribution at the cage level. For analysis of circulating peptide hormones, data below the detection limit was considered as an outlier and omitted from data analysis.

## Supplementary information

Supplementary Information_SJM

## Data Availability

All data are presented in this manuscript or supplemental figure. Transcriptomic analysis is available at NCBI GEO # GSE288998.

## References

[1] Hales, C. M., Carroll, M. D., Fryar, C. D. & Ogden, C. L. Prevalence of Obesity and Severe Obesity Among Adults: United States, 2017-2018. *NCHS Data Brief* **360**, 1–8 (2020). 32487284

[2] Pati, S., Irfan, W., Jameel, A., Ahmed, S. & Shahid, R. K. Obesity and Cancer: A Current Overview of Epidemiology, Pathogenesis, Outcomes, and Management. *Cancers (Basel)* **15**, 485 (2023). 10.3390/cancers15020485 PMC9857053 36672434

[3] Calle, E. E., Rodriguez, C., Walker-Thurmond, K. & Thun, M. J. Overweight, obesity, and mortality from cancer in a prospectively studied cohort of U.S. adults. *N. Engl. J. Med.* **348**, 1625–1638 (2003). 12711737 10.1056/NEJMoa021423

[4] Zhong, L., Liu, J., Liu, S. & Tan, G. Correlation between pancreatic cancer and metabolic syndrome: A systematic review and meta-analysis. *Front Endocrinol. ((Lausanne))* **14**, 1116582 (2023). 37113491 10.3389/fendo.2023.1116582 PMC10126301

[5] Lauby-Secretan, B. et al. Body Fatness and Cancer-Viewpoint of the IARC Working Group. *N. Engl. J. Med.* **375**, 794–798 (2016). 27557308 10.1056/NEJMsr1606602 PMC6754861

[6] Luo, J. et al. Intentional Weight Loss and Obesity-Related Cancer Risk. *JNCI Cancer Spectr.* **3**, pkz054 (2019). 31737862 10.1093/jncics/pkz054 PMC6795232

[7] Bohm, M. S. et al. The role of obesity and bariatric surgery-induced weight loss in breast cancer. *Cancer Metastasis Rev.* **41**, 673–695 (2022). 35870055 10.1007/s10555-022-10050-6 PMC9470652

[8] Koelwyn, G. J., Zhuang, X., Tammela, T., Schietinger, A. & Jones, L. W. Exercise and immunometabolic regulation in cancer. *Nat. Metab.* **2**, 849–857 (2020). 32929232 10.1038/s42255-020-00277-4 PMC9128397

[9] Look, A. R. G. et al. Intensive Weight Loss Intervention and Cancer Risk in Adults with Type 2 Diabetes: Analysis of the Look AHEAD Randomized Clinical Trial. *Obes. (Silver Spring)* **28**, 1678–1686 (2020). 10.1002/oby.22936 PMC8855671 32841523

[10] Playdon, M. C. et al. Metabolic and bariatric surgery and obesity pharmacotherapy for cancer prevention: current status and future possibilities. *J. Natl. Cancer Inst. Monogr.* **2023**, 68–76 (2023). 37139980 10.1093/jncimonographs/lgad003 PMC10157771

[11] Aminian, A. et al. Association of Bariatric Surgery With Cancer Risk and Mortality in Adults With Obesity. *JAMA* **327**, 2423–2433 (2022). 35657620 10.1001/jama.2022.9009 PMC9166218

[12] Christou, N. V., Lieberman, M., Sampalis, F. & Sampalis, J. S. Bariatric surgery reduces cancer risk in morbidly obese patients. *Surg. Obes. Relat. Dis.* **4**, 691–695 (2008). 19026373 10.1016/j.soard.2008.08.025

[13] Nierengarten, M. B. Intentional weight loss reduces cancer risk and mortality: New studies suggest that bariatric surgery can make a difference in risk and mortality reduction for obesity-related cancer types: New studies suggest that bariatric surgery can make a difference in risk and mortality reduction for obesity-related. *Cancer types Cancer* **128**, 3583–3584 (2022). 36161430 10.1002/cncr.34470

[14] Chiappetta, S. & Bottino, V. Obesity-associated cancer prevention. *Lancet Healthy Longev.* **4**, e520–e521 (2023). 37716361 10.1016/S2666-7568(23)00176-9

[15] Nogueiras, R., Nauck, M. A. & Tschop, M. H. Gut hormone co-agonists for the treatment of obesity: from bench to bedside. *Nat. Metab.* **5**, 933–944 (2023). 37308724 10.1038/s42255-023-00812-z

[16] Capozzi, M. E., DiMarchi, R. D., Tschop, M. H., Finan, B. & Campbell, J. E. Targeting the Incretin/Glucagon System With Triagonists to Treat Diabetes. *Endocr. Rev.* **39**, 719–738 (2018). 29905825 10.1210/er.2018-00117 PMC7263842

[17] Wang, L., Xu, R., Kaelber, D. C. & Berger, N. A. Glucagon-Like Peptide 1 Receptor Agonists and 13 Obesity-Associated Cancers in Patients With Type 2 Diabetes. *JAMA Netw. Open* **7**, e2421305 (2024). 38967919 10.1001/jamanetworkopen.2024.21305 PMC11227080

[18] Myers, D. J. & Wallen, J. M. in *StatPearls* (2024).

[19] Jastreboff, A. M. et al. Triple-Hormone-Receptor Agonist Retatrutide for Obesity - A Phase 2 Trial. *N. Engl. J. Med* **389**, 514–526 (2023). 37366315 10.1056/NEJMoa2301972

[20] Wilding, J. P. H. et al. Once-Weekly Semaglutide in Adults with Overweight or Obesity. *N. Engl. J. Med* **384**, 989–1002 (2021). 33567185 10.1056/NEJMoa2032183

[21] O’Flanagan, C. H., Smith, L. A., McDonell, S. B. & Hursting, S. D. When less may be more: calorie restriction and response to cancer therapy. *BMC Med* **15**, 106 (2017). 28539118 10.1186/s12916-017-0873-x PMC5442682

[22] Urva, S. et al. The novel GIP, GLP-1 and glucagon receptor agonist retatrutide delays gastric emptying. *Diab. Obes. Metab.* **25**, 2784–2788 (2023). 10.1111/dom.15167 37311727

[23] Woting, A. & Blaut, M. Small Intestinal Permeability and Gut-Transit Time Determined with Low and High Molecular Weight Fluorescein Isothiocyanate-Dextrans in C3H Mice. *Nutrients* **10**, 685 (2018). 10.3390/nu10060685 PMC6024777 29843428

[24] Inia, J. A. et al. Semaglutide Has Beneficial Effects on Non-Alcoholic Steatohepatitis in Ldlr-/-.Leiden Mice. *Int. J. Mol. Sci.* **24**, 8494 (2023). 10.3390/ijms24108494 PMC10218334 37239841

[25] Zhu, R. & Chen, S. Proteomic analysis reveals semaglutide impacts lipogenic protein expression in epididymal adipose tissue of obese mice. *Front Endocrinol. ((Lausanne))* **14**, 1095432 (2023). 37025414 10.3389/fendo.2023.1095432 PMC10070826

[26] Liu, D. et al. Comparison of Beneficial Metabolic Effects of Liraglutide and Semaglutide in Male C57BL/6J Mice. *Can. J. Diab.* **46**, 216–224 e212 (2022). 10.1016/j.jcjd.2021.08.012 35568421

[27] Liu, X. et al. Calorie restriction and calorie dilution have different impacts on body fat, metabolism, behavior, and hypothalamic gene expression. *Cell Rep.* **39**, 110835 (2022). 35584669 10.1016/j.celrep.2022.110835

[28] McGrath, C. et al. Exercise Degrades Bone in Caloric Restriction, Despite Suppression of Marrow Adipose Tissue (MAT). *J. Bone Min. Res* **35**, 106–115 (2020). 10.1002/jbmr.3872 PMC6980282 31509274

[29] Engber, D. What models eat. *Nat. Med* **24**, 692–695 (2018). 29875464 10.1038/s41591-018-0055-1

[30] Li, J. et al. Tumor Cell-Intrinsic Factors Underlie Heterogeneity of Immune Cell Infiltration and Response to Immunotherapy. *Immunity* **49**, 178–193 e177 (2018). 29958801 10.1016/j.immuni.2018.06.006 PMC6707727

[31] Sodhi, M., Rezaeianzadeh, R., Kezouh, A. & Etminan, M. Risk of Gastrointestinal Adverse Events Associated With Glucagon-Like Peptide-1 Receptor Agonists for Weight Loss. *JAMA* **330**, 1795–1797 (2023). 37796527 10.1001/jama.2023.19574 PMC10557026

[32] Coskun, T. et al. LY3437943, a novel triple glucagon, GIP, and GLP-1 receptor agonist for glycemic control and weight loss: From discovery to clinical proof of concept. *Cell Metab.* **34**, 1234–1247 e1239 (2022). 35985340 10.1016/j.cmet.2022.07.013

[33] Bain, S. C. & Min, T. A new class of glucose-lowering therapy for type 2 diabetes: the latest development in the incretin arena. *Lancet* **402**, 504–505 (2023). 37385276 10.1016/S0140-6736(23)01182-0

[34] Su, K. Z. et al. Relation of Circulating Resistin to Insulin Resistance in Type 2 Diabetes and Obesity: A Systematic Review and Meta-Analysis. *Front Physiol.* **10**, 1399 (2019). 31803062 10.3389/fphys.2019.01399 PMC6877503

[35] Himbert, C. et al. Signals from the Adipose Microenvironment and the Obesity-Cancer Link-A Systematic Review. *Cancer Prev. Res ((Philos.))* **10**, 494–506 (2017). 10.1158/1940-6207.CAPR-16-0322 PMC5898450 28864539

[36] Jacobson, S. et al. Hyperglycemia as a risk factor in pancreatic cancer: A nested case-control study using prediagnostic blood glucose levels. *Pancreatology* **21**, 1112–1118 (2021). 10.1016/j.pan.2021.05.008 34049822

[37] Pang, Y. et al. Associations of diabetes, circulating protein biomarkers, and risk of pancreatic cancer. *Br. J. Cancer* **130**, 504–510 (2024). 38129526 10.1038/s41416-023-02533-2 PMC10844301

[38] Abdullah Bin Ahmed, I. A Comprehensive Review on Weight Gain following Discontinuation of Glucagon-Like Peptide-1 Receptor Agonists for Obesity. *J. Obes.* **2024**, 8056440 (2024). 38765635 10.1155/2024/8056440 PMC11101251

[39] Han, M. S. et al. Regulation of adipose tissue inflammation by interleukin 6. *Proc. Natl. Acad. Sci. USA* **117**, 2751–2760 (2020). 31980524 10.1073/pnas.1920004117 PMC7022151

[40] Minafra, A. R. et al. Interleukin 6 receptor is not directly involved in regulation of body weight in diet-induced obesity with and without physical exercise. *Front Endocrinol. ((Lausanne))* **13**, 1028808 (2022). 36387898 10.3389/fendo.2022.1028808 PMC9647089

[41] Khan, M. S. et al. Interleukin-6 and Cardiovascular Events in Healthy Adults. *Mesa. JACC Adv.* **3**, 101063 (2024). 39077632 10.1016/j.jacadv.2024.101063 PMC11284704

[42] Timper, K. et al. IL-6 Improves Energy and Glucose Homeostasis in Obesity via Enhanced Central IL-6 trans-Signaling. *Cell Rep.* **19**, 267–280 (2017). 28402851 10.1016/j.celrep.2017.03.043

[43] Fuster, J. J. & Walsh, K. The good, the bad, and the ugly of interleukin-6 signaling. *EMBO J.* **33**, 1425–1427 (2014). 24850773 10.15252/embj.201488856 PMC4194086

[44] Orange, S. T., Leslie, J., Ross, M., Mann, D. A. & Wackerhage, H. The exercise IL-6 enigma in cancer. *Trends Endocrinol. Metab.* **34**, 749–763 (2023). 37633799 10.1016/j.tem.2023.08.001

[45] Wang, L., Wang, W., Kaelber, D. C., Xu, R. & Berger, N. A. GLP-1 Receptor Agonists and Colorectal Cancer Risk in Drug-Naive Patients With Type 2 Diabetes, With and Without Overweight/Obesity. *JAMA Oncol.* **10**, 256–258 (2024). 38060218 10.1001/jamaoncol.2023.5573 PMC10704339

[46] Zhu, C. et al. Comprehensively prognostic and immunological analyses of GLP-1 signaling-related genes in pan-cancer and validation in colorectal cancer. *Front Pharm.* **15**, 1387243 (2024). 10.3389/fphar.2024.1387243 PMC11298396 39104385

[47] Bezin, J. et al. GLP-1 Receptor Agonists and the Risk of Thyroid Cancer. *Diab. Care* **46**, 384–390 (2023). 10.2337/dc22-1148 36356111

[48] Silverii, G. A. et al. Glucagon-like peptide-1 receptor agonists and risk of thyroid cancer: A systematic review and meta-analysis of randomized controlled trials. *Diab. Obes. Metab.* **26**, 891–900 (2024). 10.1111/dom.15382 38018310

[49] Pasternak, B. et al. Glucagon-like peptide 1 receptor agonist use and risk of thyroid cancer: Scandinavian cohort study. *BMJ* **385**, e078225 (2024). 38683947 10.1136/bmj-2023-078225 PMC11004669

[50] Hu, W. et al. Use of GLP-1 Receptor Agonists and Occurrence of Thyroid Disorders: a Meta-Analysis of Randomized Controlled Trials. *Front Endocrinol. ((Lausanne))* **13**, 927859 (2022). 35898463 10.3389/fendo.2022.927859 PMC9309474

[51] Chaib, M. et al. Protein kinase C delta regulates mononuclear phagocytes and hinders response to immunotherapy in cancer. *Sci. Adv.* **9**, eadd3231 (2023). 38134280 10.1126/sciadv.add3231 PMC10745701

[52] Sipe, L. M. et al. Response to immune checkpoint blockade improved in pre-clinical model of breast cancer after bariatric surgery. *Elife* **11**, e79143 (2022). 10.7554/eLife.79143 PMC9342954 35775614

[53] Nagy, C. & Einwallner, E. Study of In Vivo Glucose Metabolism in High-fat Diet-fed Mice Using Oral Glucose Tolerance Test (OGTT) and Insulin Tolerance Test (ITT). *J. Vis. Exp.*10.3791/56672 (2018). 10.3791/56672 PMC5908452 29364280

[54] Freemerman, A. J. et al. Myeloid Slc2a1-Deficient Murine Model Revealed Macrophage Activation and Metabolic Phenotype Are Fueled by GLUT1. *J. Immunol.* **202**, 1265–1286 (2019). 30659108 10.4049/jimmunol.1800002 PMC6360258

[55] Ewels, P. A. et al. The nf-core framework for community-curated bioinformatics pipelines. *Nat. Biotechnol.* **38**, 276–278 (2020). 32055031 10.1038/s41587-020-0439-x

[56] Love, M. I., Anders, S., Kim, V. & Huber, W. RNA-Seq workflow: gene-level exploratory analysis and differential expression. *F1000Res* **4**, 1070 (2015). 26674615 10.12688/f1000research.7035.1 PMC4670015

[57] Love, M. I., Huber, W. & Anders, S. Moderated estimation of fold change and dispersion for RNA-seq data with DESeq2. *Genome Biol.* **15**, 550 (2014). 25516281 10.1186/s13059-014-0550-8 PMC4302049

[58] Subramanian, A. et al. Gene set enrichment analysis: a knowledge-based approach for interpreting genome-wide expression profiles. *Proc. Natl. Acad. Sci. USA* **102**, 15545–15550 (2005). 16199517 10.1073/pnas.0506580102 PMC1239896

[59] Castanza, A. S. et al. Extending support for mouse data in the Molecular Signatures Database (MSigDB). *Nat. Methods* **20**, 1619–1620 (2023). 37704782 10.1038/s41592-023-02014-7 PMC11397807

[60] Chaib, M. et al. PKC agonism restricts innate immune suppression, promotes antigen cross-presentation and synergizes with agonistic CD40 antibody therapy to activate CD8(+) T cells in breast cancer. *Cancer Lett.* **531**, 98–108 (2022). 35074498 10.1016/j.canlet.2022.01.017 PMC9867936

