# Supplementary material for: Incretin triple agonist retatrutide (LY3437943) alleviates obesity-associated cancer progression
Source: NPJ Metab Health Dis. 2025 Mar 14;3:10. doi: 10.1038/s44324-025-00054-5 (PMC11908972; doi:10.1038/s44324-025-00054-5)
Supplement: Supplementary file 1 — Supplementary Information_SJM [file 44324_2025_54_MOESM1_ESM.pdf]

Supplementary Information

Incretin triple agonist retatrutide (LY3437943) alleviates obesity-associated cancer progression

Sandesh J. Marathe[1,2], Emily W. Grey[1], Margaret S. Bohm[3], Sydney C. Joseph[1], Arvind V. Ramesh[1], Matthew A. Cottam[4], Kamran Idrees[4], Kathryn E. Wellen[5], Alyssa H. Hasty[6,7,8,9], Jeffrey C. Rathmell[7,10], Liza Makowski[1,2,3]*

Supplementary Fig. 1.

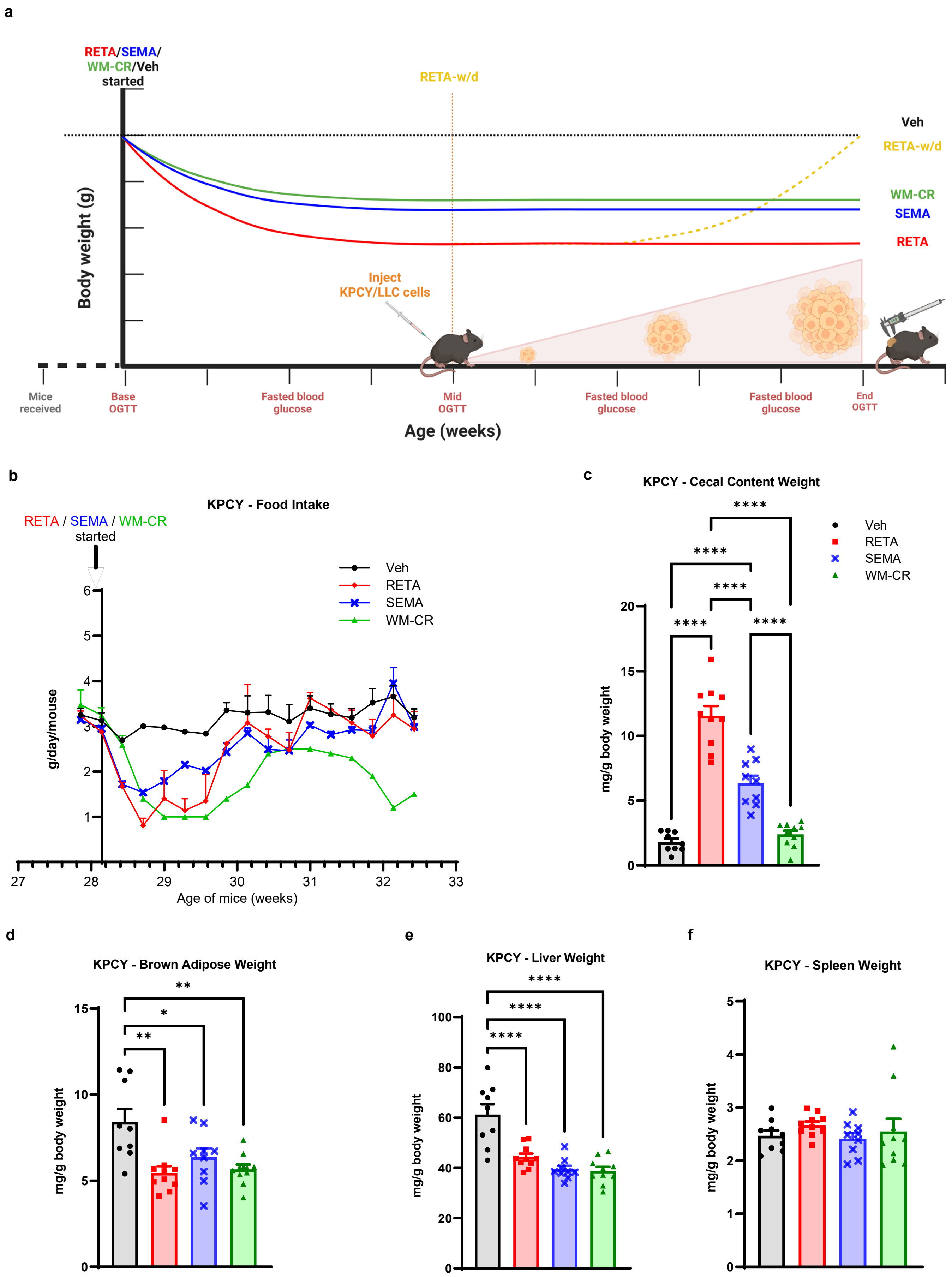

Supplementary Fig. 1: RETA and SEMA impact food intake and delay gastric emptying, reduce brown adipose tissue and liver weights, but do not affect spleen weights in the KPCY model. a, Study design. Diet-induced obese (DIO) male C57BL/6J mice were purchased at 16 weeks of age and acclimated for three weeks before initiating the study. Mice were maintained on an obesogenic high fat diet throughout the study. Vehicle "Veh" control or retatrutide "RETA" (30 nmol/kg on alternate days, subcutaneous, SQ) or semaglutide "SEMA" (30 nmol/kg on alternate days, SQ) or caloric restriction for weight matched to SEMA "WM-CR" was initiated. After 2 weeks of treatment, RETA treatment was switched to Veh treatment for the RETA withdrawal (RETA-w/d) group. KPCY 2838 or LLC cancer cells were injected into the right flank of all mice. Oral glucose tolerance test (OGTT) for KPCY – RETA-w/d model and fasted blood glucose were recorded as indicated. **b**, Food intake was measured daily, and data are represented as mean food intake binned for 3 days. **c**, Cecal contents were weighed at endpoint and normalized to total body weight. **d**, Brown adipose tissue (BAT), **e**, liver and **f**, spleen weights normalized to body weights were quantified at endpoint. Data is represented as mean ± SEM (N=9 mice Veh, N=10 mice RETA, N=9 mice SEMA, N=10 mice WM-CR). **c-f**, Statistical significance was determined by one-way ANOVA with Tukey's MCT and is denoted as $*P \leq 0.05$, $**P \leq 0.01$, $****P \leq 0.0001$.

Supplementary Fig. 2.

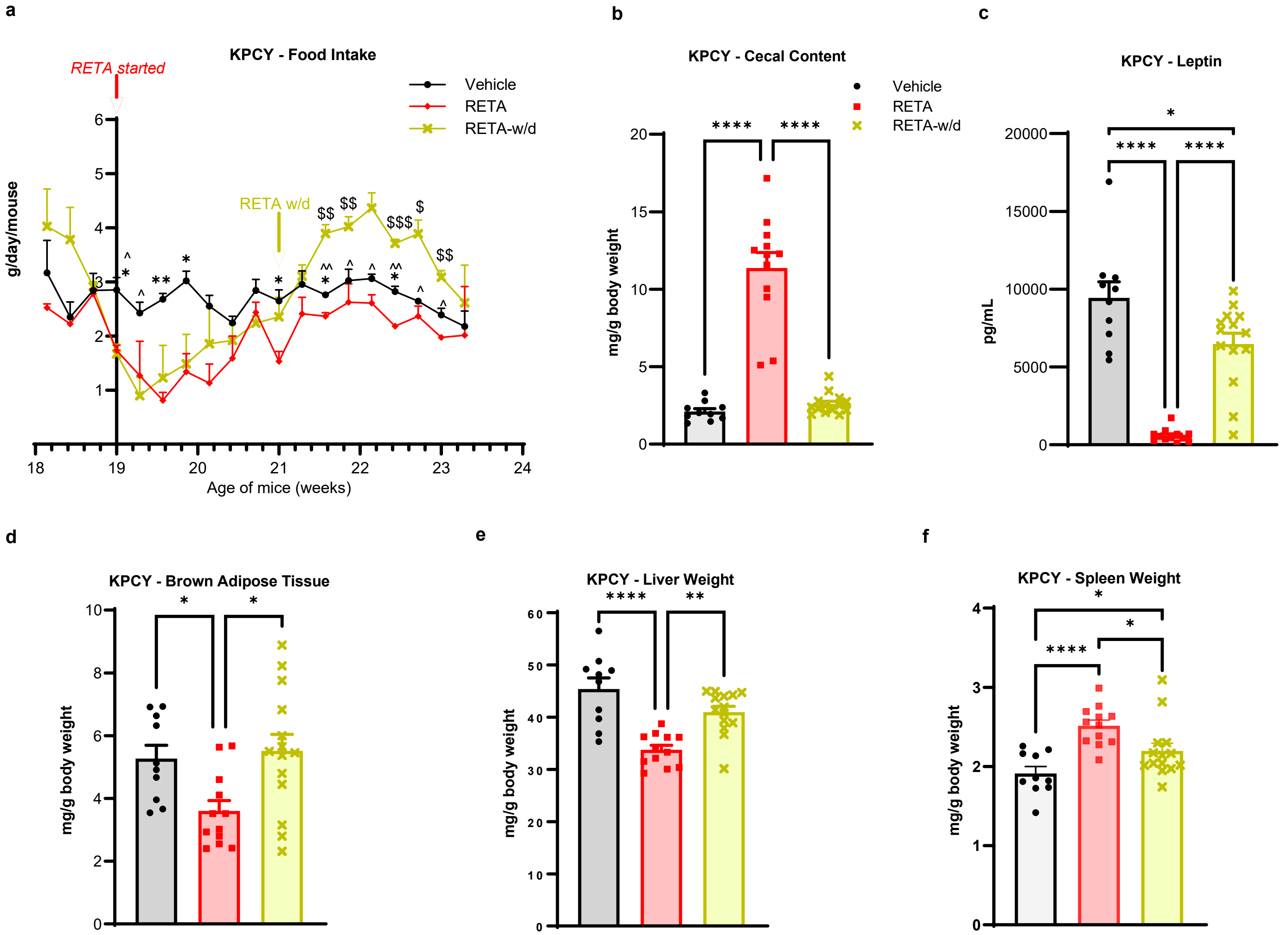

Supplementary Fig. 2: RETA withdrawal restores food intake and gastric motility, reverses the effects on leptin concentrations, brown adipose tissue, liver, and spleen weights in the KPCY model. a, Food intake was measured daily, and data are represented as mean food intake binned for 3 days. **b**, Cecal contents were weighed at endpoint and normalized to total body weight. **c**, Plasma concentrations of leptin are reported. **d**, Brown adipose tissue (BAT), **e**, liver and **f**, spleen weights normalized to body weights were quantified at endpoint. Data is represented as mean ± SEM (N=10 mice Veh, N=12 mice RETA, N=14 mice RETA-w/d). **a**, Statistical significance was determined by two-way ANOVA with repeated measures using a mixed-effects model and Tukey's multiple comparison test. $*P \leq 0.05$, $**P \leq 0.01$, Veh versus RETA; $\hat{}P \leq 0.05$, $\hat{}\hat{}P \leq 0.01$, Veh versus RETA-w/d; $\$P \leq 0.05$, $\$\$P \leq 0.01$, $\$\$\$P \leq 0.001$, RETA versus RETA-w/d. **b-f**, Statistical significance was computed using one-way ANOVA with Tukey's test and is denoted as $*P \leq 0.05$; $**P \leq 0.01$; $****P \leq 0.0001$.

Supplementary Fig. 3.

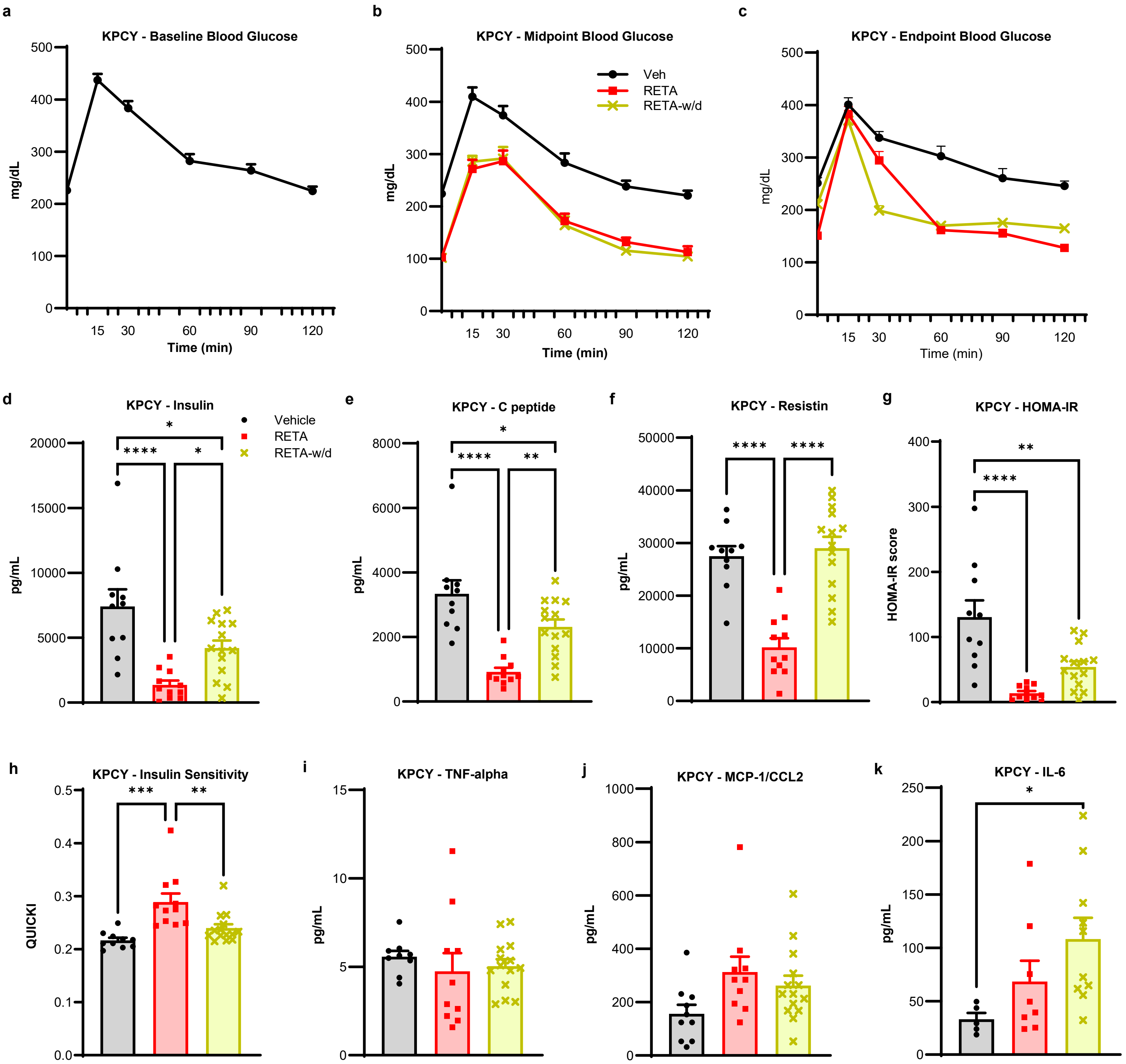

Supplementary Fig. 3: RETA withdrawal partially rescinds insulin concentration and resistance, and significantly increases IL-6 concentrations in the KPCY model. OGTT was performed at **a**, baseline, **b**, midpoint, and **c**, endpoint. Fasted plasma **d**, insulin and **e**, C-peptide concentrations were measured at endpoint. **f**, Fasted plasma resistin concentrations at endpoint are reported. **g**, Insulin resistance was calculated by Homeostatic Model Assessment for Insulin Resistance (HOMA-IR) score. **h**, The quantitative insulin sensitivity check index (QUICKI) was calculated. Plasma concentrations of **i**, TNF-alpha, **j**, MCP-1/CCL2, and **k**, IL-6 in KPCY model are reported. Data is represented as mean ± SEM (**a-h**, N=10 mice Veh, N=12 mice RETA, N=14 mice RETA-w/d; **i-k**, N=6-10 mice Veh, N=9-12 mice RETA, N=10-14 mice RETA-w/d)). Statistical significance was computed using one-way ANOVA with Tukey's test and is denoted as $*P \leq 0.05$; $**P \leq 0.01$; $***P \leq 0.001$; $****P \leq 0.0001$.

Supplementary Fig. 4.

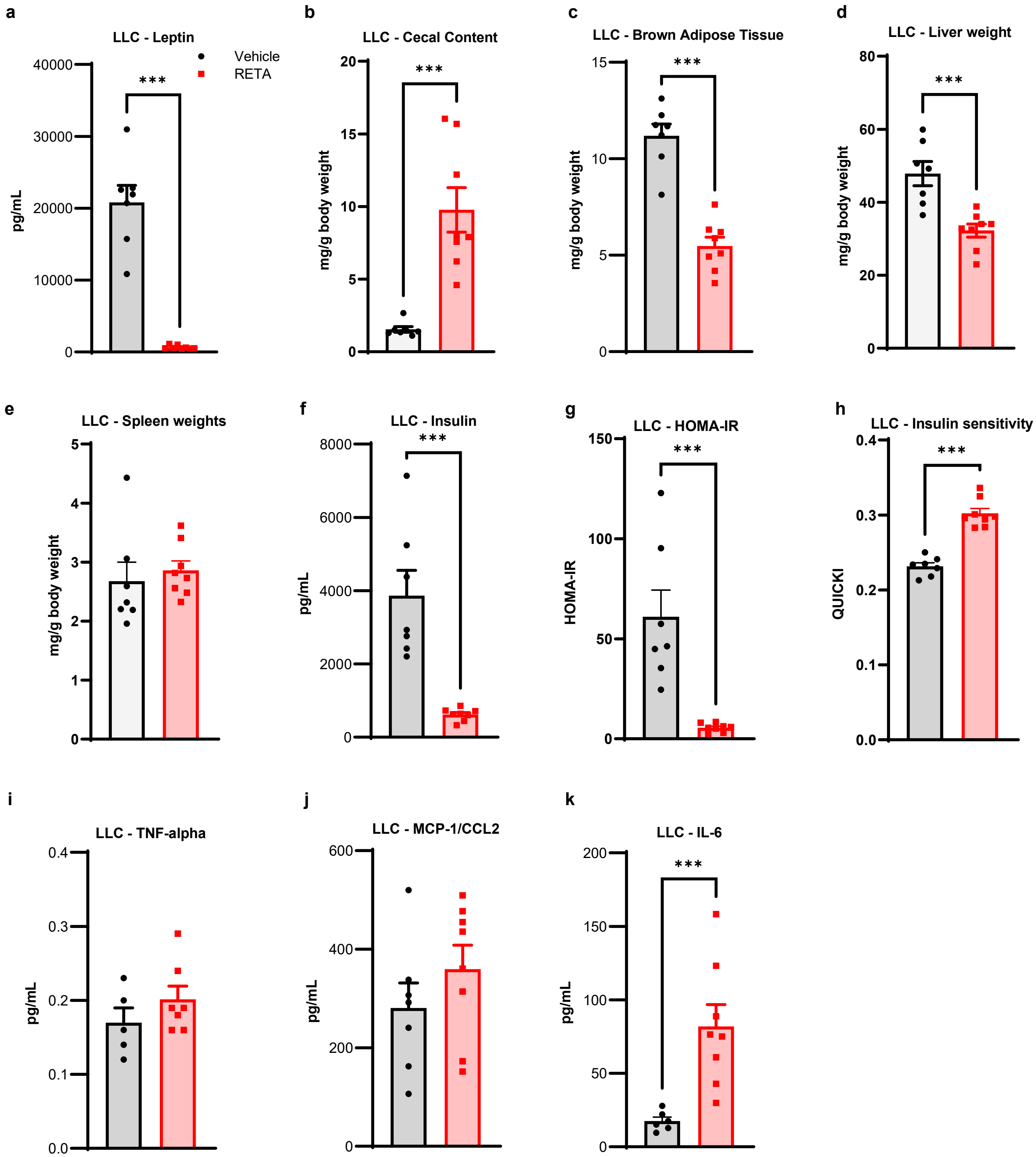

Supplementary Fig. 4: RETA treatment significantly reduces leptin levels, gastric motility, brown adipose tissue and liver weights, insulin levels and resistance, while significantly increasing IL-6 levels in the LLC model. a, Plasma leptin concentrations at endpoint are reported. **b**, Cecal contents, **c**, brown adipose tissue, **d**, liver, **e**, spleen weights normalized to body weights at endpoint are reported. **f**, Fasted plasma insulin concentrations were measured at endpoint. **g**, Insulin resistance was calculated by Homeostatic Model Assessment for Insulin Resistance (HOMA-IR) score. **h**, The quantitative insulin sensitivity check index (QUICKI) was calculated. Plasma concentrations of **i**, TNF-alpha, **j**, MCP-1/CCL2, and **k**, IL-6, in LLC model are reported. Data are represented as mean ± SEM (N=5-7 Veh, N=7-8 RETA). Statistical significance was determined by Student's *t*-test with Mann-Whitney test and is denoted as ***$P \leq 0.001$.

a. Myeloid panel

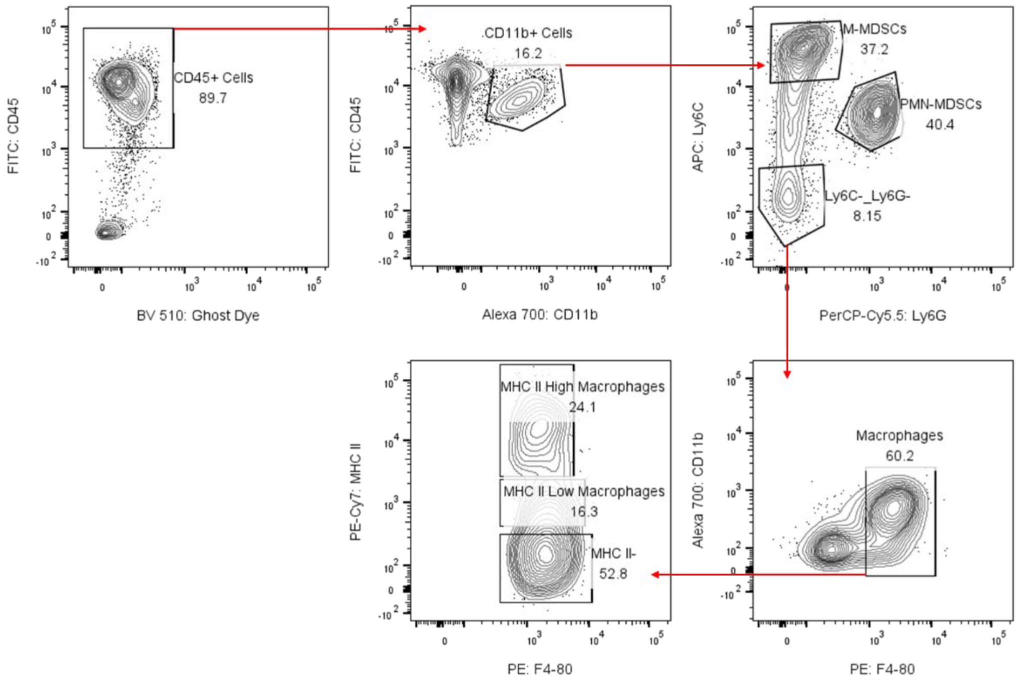

b. T cell panel

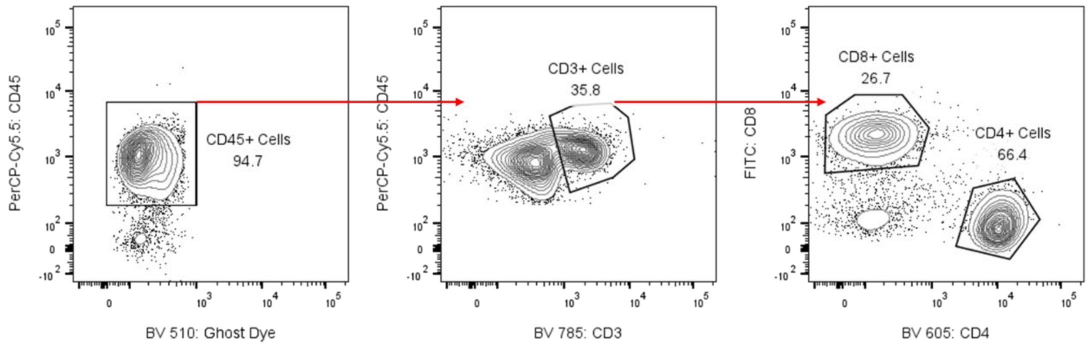

Supplementary Fig. 5: Gating schemata for Flow cytometry drawn in FlowJo version 10 software

The ARRIVE Essential 10: Compliance Questionnaire

Use this questionnaire to evaluate how well a manuscript complies with the ARRIVE Essential 10. It can be applied to any manuscript describing comparative experiments in living animals, by assessors such as journal staff, editors, or peer reviewers.

Item		Question(s)	Answers
1	Study Design	Are all experimental and control groups clearly identified?	☒ Yes, for at least one experiment<br>☐ No
		Is the experimental unit (e.g. an animal, litter or cage of animals) clearly identified?	☒ Yes, for at least one experiment<br>☐ No
2	Sample Size	Is the exact number of experimental units in each group at the start of the study provided (e.g. in the format '*n*=')?	☒ Yes, for at least one experiment<br>☐ No
		Is the method by which the sample size was chosen explained?	☐ Yes, for at least one experiment<br>☒ No
3	Inclusion & Exclusion Criteria	Are the criteria used for including and excluding animals, experimental units, or data points provided?	☐ Yes, for at least one experiment<br>☒ No
		Are any exclusions of animals, experimental units, or data points reported, or is there a statement indicating that there were no exclusions?	☒ Yes, for at least one analysis<br>☐ No
4	Randomisation	Is the method by which experimental units were allocated to control and treatment groups described?	☐ Yes, for at least one experiment<br>☒ No
5	Blinding	Is it clear whether researchers were aware of, or blinded to, the group allocation at any stage of the experiment or data analysis?	☐ Yes, for at least one experiment<br>☒ No
6	Outcome Measures	For all experimental outcomes presented, are details provided of exactly what parameter was measured?	☒ Yes, for at least one experiment<br>☐ No
7	Statistical Methods	Is the statistical approach used to analyse each outcome detailed?	☒ Yes, for at least one analysis<br>☐ No
		Is there a description of any methods used to assess whether data met statistical assumptions?	☐ Yes, for at least one analysis<br>☐ No<br>☒ Not applicable
8	Experimental Animals	Are all species of animal used specified?	☒ Yes, for at least one experiment<br>☐ No
		Is the sex of the animals specified?	☒ Yes, for at least one experiment<br>☐ No<br>☐ Not applicable to species
		Is at least one of age, weight or developmental stage of the animals specified?	☒ Yes, for at least one experiment<br>☐ No
9	Experimental Procedures	Are both the timing and frequency with which procedures took place specified?	☒ Yes, for at least one experiment<br>☐ No
		Are details of acclimatisation periods to experimental locations provided?	☒ Yes, for at least one experiment<br>☐ No
10	Results	Are descriptive statistics for each experimental group provided, with a measure of variability (e.g. mean and SD, or median and range)?	☒ Yes, for at least one experiment<br>☐ No<br>☐ Not applicable to the type of data collected
		Is the effect size and confidence interval provided?	☒ Yes, for at least one experiment<br>☐ No<br>☐ Not applicable to the type of analysis used
